# Development of “-omics” research in *Schistosoma* spp. and -omics-based new diagnostic tools for schistosomiasis

**DOI:** 10.3389/fmicb.2014.00313

**Published:** 2014-06-26

**Authors:** Shuqi Wang, Wei Hu

**Affiliations:** ^1^Department of Microbiology and Microbial Engineering, School of Life Sciences, Fudan UniversityShanghai, China; ^2^Key Laboratory of Parasite and Vector Biology of Ministry of Health, National Institute of Parasitic Diseases, Chinese Center for Diseases Control and PreventionShanghai, China

**Keywords:** *Schistosoma*, -omics, diagnosis, biomarkers, parasite

## Abstract

Schistosomiasis, caused by dioecious flatworms in the genus *Schistosoma*, is torturing people from many developing countries nowadays and frequently leads to severe morbidity and mortality of the patients. Praziquantel based chemotherapy and morbidity control for this disease adopted currently necessitate viable and efficient diagnostic technologies. Fortunately, those “-omics” researches, which rely on high-throughput experimental technologies to produce massive amounts of informative data, have substantially contributed to the exploitation and innovation of diagnostic tools of schistosomiasis. In its first section, this review provides a concise conclusion on the progresses pertaining to schistosomal “-omics” researches to date, followed by a comprehensive section on the diagnostic methods of schistosomiasis, especially those innovative ones based on the detection of antibodies, antigens, nucleic acids, and metabolites with a focus on those achievements inspired by “-omics” researches. Finally, suggestions about the design of future diagnostic tools of schistosomiasis are proposed, in order to better harness those data produced by “-omics” studies.

Schistosomiasis or “Bilharziasis” refers to the parasitic diseases caused by dioecious flatworms in the genus *Schistosoma.* The main pathogenic species of human schistosomiasis comprise *Schistosoma mansoni*, *S. japonicum*, and *S. haematobium*. Free living larvae of these pathogens released by variant kinds of snails in the fresh water, i.e., the cercariae can penetrate the skin of their definite hosts and subsequently bring about either egg-induced chronic responses, known as intestinal schistosomiasis (*S. mansoni and S. japonicum*) and urinary schistosomiasis (*S. haematobium*; Hu et al., 2004), or, less frequently, some acute reactions like toxemia or cytokine-induced shock (Wynn et al., 2004). These highly debilitating symptoms account for the severe morbidity and mortality incurred by schistosomiasis and render it a key poverty contributor for some developing countries in the tropical and subtropical regions. Moreover, schistosomiasis has also become an emerging threat in non-endemic areas due to the mounting numbers of immigrants and tourists (Patz et al., 2000; Enk et al., 2010). Currently, praziquantel (PZQ) based chemotherapy and morbidity control have become the predominantly adopted strategy for the treatment and control of schistosomiasis worldwide (Qing-Wu et al., 2002; Zhou et al., 2011b). Consequently, monitoring disease transmission, seeking patients for treatment in endemic areas and the evaluation of remedies entail viable and efficient diagnostic technologies, in order to optimize the extant control and prevention project for schistosomiasis (Balog et al., 2010).

## STATUS QUO OF THE CURRENT SCHISTOSOMAL “-Omics” RESEARCHES

“-omics” is a suffix labeling a series of biological subjects that adopt high-throughput experimental technologies to produce a large body of holistic and informative data. Since the launching of the* Schistosoma* Genome Project propelled by the WHO in 1994 (Oliveira et al., 2008), colossal progresses have been made in various *Schistosoma* -omics arenas including genomics, transcriptomics, proteomics, metabonomics (Ju et al., 2010), to name but a few. Besides an augmented understanding of schistosome biology and pathogenesis, those knowledge acquired by *Schistosoma* -omics researches also substantially contribute to the exploitation and innovation of drugs, vaccines as well as diagnostic tools for schistosomiasis. This review aims to display the representative achievements of those schistosomal -omics researches and conclude the diagnostic inventions derived from or at least inspired by those -omics studies.

### TRANSCRIPOMICS

*Schistosoma* transcriptomics research commenced from the establishment of complementary DNA (cDNA) libraries which generated expressed sequence tags (ESTs) from nearly all stages of *S. mansoni* and *S. japonicum* (Fan et al., 1998; Fung et al., 2002; Hu et al., 2003; Peng et al., 2003; Shabaan et al., 2003; Verjovski-Almeida et al., 2003; Merrick et al., 2009). After the obtainment of numerous ESTs data, microarray and serial analysis of gene expression (SAGE) technology have been used to profile the transcripts of schistosomes in different stages and/or under distinct conditions. In particular, Geoffrey Gobert classified these transcriptomics applications into four main categories (Gobert, 2010), i.e., (a) characterizing individual cell/tissue types, (Jones et al., 2007; Gobert et al., 2009a), (b) profiling the intact organism and lifecycle (Hoffmann et al., 2002; Fitzpatrick et al., 2004, 2005, 2009; Hoffmann and Fitzpatrick, 2004; Chai et al., 2006; Dillon et al., 2006, 2008; Gobert et al., 2006, 2009b; Moertel et al., 2006; Vermeire et al., 2006; Jolly et al., 2007; Ojopi et al., 2007; Williams et al., 2007; Hu et al., 2009; Taft et al., 2009), (c) parasite–host interactions and effect of therapies on parasite (Hoffmann et al., 2001; Alger and Williams, 2002; Sandler et al., 2003; Aragon et al., 2008, 2009; Gobert and Jones, 2008; de Moraes Mourão et al., 2009; You et al., 2009; Burke et al., 2010; Gobert et al., 2010), and (d) gene expression differences between geographical isolates or species (Le et al., 2002; Fitzpatrick et al., 2004; Moertel et al., 2006). Not until 2012 was the transcriptome of the adult and egg stages of *S. haematobium* profiled along with its genome (Young et al., 2012).

### MicroRNA (miRNA) GENOMICS

Unlike messenger RNAs (mRNAs), small non-coding RNAs (sncRNAs) represent a group of untranslatable transcripts which are approximately 18–30 nt in length and serve as critical regulators to silence or activate specific target genes in a variety of organisms (Bartel, 2004; Molnar et al., 2010). Endogenous-small interfering (Endo-siRNAs), miRNAs, and Piwi-interacting RNA (piRNAs) are three main components of sncRNAs (Kim, 2005). Using protocols similar to the conventional transcriptomic researches, which can be outlined as RNA isolation, library construction, and sequencing (Cheng and Jin, 2012), vast numbers of schistosomal miRNAs and endo-siRNAs have been successfully detected in *S. mansoni* (Copeland et al., 2009; de Souza Gomes et al., 2011; Simões et al., 2011) and *S. japonicum* (Xue et al., 2008; Copeland et al., 2009; Hao et al., 2010; Wang et al., 2010b; Cai et al., 2011). What’s more, recently Cai et al. (2012) adopted a totally novel method, i.e., the immunoprecipitation of *Sj*Ago2, a key factor in sncRNAs biogenesis with monoclonal antibodies (mAbs) to identify and characterize the associated small RNAs. Further classification steps showed that endo-siRNAs derived from transposable elements (TEs) were prominent among those conjugated sncRNAs.

### PROTEOMICS

Since the correlation between transcriptional level and translational level of one expressed gene is not necessarily straightforward, proteomic analyses are also of great importance aiming at the characterization and comparison of functions, abundance, and subcellular localization (Ju et al., 2010) of numerous gene products derived from single or multiple samples. In a typical proteomics research, schistosomal specimen would be separated by one dimensional-(1D-) or 2D-polyacrylamide gel electrophoresis (PAGE) in the beginning. Subsequently, the isolated bands or spots would be subjected to digestion and either Matrix-assisted laser desorption/ionization time-of-flight mass spectrometer (MALDI-TOF-MS) or liquid chromatography-tandem mass spectrometry (LC-MS/MS) according to the separation method. Finally, protein identities within the sample would be acquired through comparing the mass spectrometry (MS) results to the theoretical masses stocked in a particular database (van Hellemond et al., 2007). Based on the purpose of proteomics researches, schistosomal samples ought to be prepared in different ways. So far, schistosomal proteomics has been applied to the investigation and comparison of protein compositions in various developmental stages (Curwen et al., 2004; Liu et al., 2006) or between different genders (Cheng et al., 2005; Liu et al., 2006) and the worm proteins might be pre-fractioned accordingly, e.g., soluble membrane protein (Curwen et al., 2004; Cheng et al., 2005), tegumental fractions (Van Balkom et al., 2005; Braschi et al., 2006; Braschi and Wilson, 2006; Liu et al., 2006; Mulvenna et al., 2010; Castro-Borges et al., 2011), secreted antigens (Knudsen et al., 2005; Curwen et al., 2006; Liu et al., 2009), gut contents (Delcroix et al., 2007), etc.

### IMMUNOMICS

Immunomics is a study that combines proteomics with serology and aims at ascertaining the interaction between host immune system and exotic antigens after pathogen invasion. Early schistosomal immunomics work utilized combined 2D Western blotting and MS to identify and compare proteins recognized by serum samples from *S. haematobium* exposed patients before and after PZQ treatment (Mutapi et al., 2005) or with different ages and infection intensities (Mutapi et al., 2008). Afterwards, an immunomics protein microarray technology was also applied in an attempt to seek prospective vaccine targets (Driguez et al., 2010). This advanced technology consists of several steps including genes selection, cell-free expression, chip printing, and serological probing.

### GLYCOMICS

In view of the critical role glycans played in the induction of innate immune responses during schistosome–host interplay, glycomics, the profiling of the schistosomal protein- and lipid-conjugated glycan or glycan elements was addressed even before the screening of schistosomal transcriptomics and proteomics (Hokke et al., 2007). So far, the patterns of glycoconjugates expressed by multiple life stages of *S. mansoni* have been precisely elucidated by either anticarbohydrate mAbs identification or MS-based method (Khoo et al., 1995, 1997a,b, 2001; van Remoortere et al., 2000, 2003; Wuhrer et al., 2000, 2002, 2006a,b; Huang et al., 2001; Nyame et al., 2003; Robijn et al., 2005, 2007a,b).

### GENOMICS

Under the continual efforts from two consortia of researchers, the genome of* S. mansoni* and *S. japonicum* were deciphered and published simultaneously in 2009 (Berriman et al., 2009; The *Schistosoma japonicum* Genome Sequencing and Functional Analysis Consortium, 2009). Three years later, the genome information of *S. haematobium*, the pathogen causing urogenital schistosomiasis also became accessible (Young et al., 2012). Overall, the genome of the preceding three main causative human schistosomiasis pathogens are organized into eight chromosomes, including seven pairs of autosomes and a pair of ZW type sex chromosomes. The genome of *S. mansoni* (363 Mb), *S. japonicum* (397 Mb), and *S. haematobium* (385 Mb) encompass 13,184, 13,469, and 13,073 protein-coding genes, respectively, and a considerable proportion of them were mapped into gene ontology (GO) categories. Apart form those protein coding genes, more than 40% of these three genomes are taken up by repetitive elements, which contains retrotransposons, satellites, DNA transposons and other kinds of unknown repeats. As a whole, the relationship between *S. mansoni* and *S. haematobium* are more intimate than either of them with *S. japonicum* (Young et al., 2012).

### METABONOMICS

Schistosomes-related metabonomics keeps tabs on the overall changes of metabolites within tissues and biofluids of the hosts before and after blood fluke infection. Metabonomics-based schistosomiasis biomarker discoveries were pioneered in 2004 by Wang et al. (2004), who used ^1^H nuclear magnetic resonance (NMR) spectroscopy and multivariate pattern recognition to analyze the metabolic signature of urine samples from *S. mansoni* infected mice. Subsequently, using various spectroscopic methods, e.g., NMR spectroscopy, MS, and capillary electrophoresis (CE), coupled with various data-mining technologies (Legido-Quigley, 2010), several metabonomics related to schistosome infection were profiled, such as urine samples from mice infected with *S. mansoni* (Garcia-Perez et al., 2008, 2009, 2010) and hamsters infected with *S. japonicum* (Wang et al., 2006), as well as blood and multiple tissue samples from schistosome-alone infected (Li et al., 2009) or two pathogens co-infected rodents (Wu et al., 2010). It was not until early this decade that one metabonomic investigation started to survey human *Schistosoma* spp. infection (Balog et al., 2011). Corresponding works will be reviewed in the next section.

## THE “NEW GENERATION” DIAGNOSTIC METHODS FOR SCHISTOSOMIASIS AND THEIR PROGRESSES IMPELLED BY THE “-OMICS” ACHIEVEMENTS

### TRADITIONAL, ETIOLOGICAL DIAGNOSTIC METHODS

Etiological diagnosis of schistosomiasis, i.e., the microscopic detection of schistosome eggs in urine (*S. haematobium*) or stool (*S. mansoni* and *S. japonicum*) samples is the most straightforward and widely adopted diagnostic approach to investigate the infection of schistosomes (Utzinger et al., 2011). Representative methods of etiological diagnosis include Kato–Katz (KK) thick smear (Katz et al., 1972), miracidium hatching assay (Jurberg et al., 2008), egg floating technique (FLOTAC; Cringoli, 2006), nuclepore urine filtration (Mott et al., 1982), etc. Those etiological methods are deemed as the “gold standard” for the detection of cases particularly in high endemicity settings and in the early stage of a control program. However, after mass drug administration or in low transmission settings, these traditional methods show limited sensitivity and accuracy (Zhao et al., 2012). In addition, multiple stool examinations or combined techniques, although reduced the false-negative rate to some extent, are quite time-consuming and labor-intensive, which hindered their application in large-scale epidemiological surveys (Zhou et al., 2011b). Thus, they have been gradually replaced by immunological techniques based on antigen or antibody detection, as well as PCR-based molecular tools, metabolic profiling, i.e., those so-called “new generation” diagnostic methods. Meanwhile, those novel diagnostic methods have benefited continuously from the previously introduced “-omics” researches (**Table 1**).

**Table 1:** An overview of “-omics” researches’ contribution to the revelation of novel schistosomiasis diagnostic targets.

Diagnostic techniques	“-omics” researches		Representative diagnostic targets
Antibody-based immunodiagnosis	Transcriptomics	Fung et al. (2002)	Sjp40 (Zhou et al., 2010)
		Wu et al. (1998)	Sj13 (Zhou et al., 2009)
		Hu et al. (2003)	*Sj*EFCAB (Lu et al., 2012a), SjE16 (Wang et al., 2003), *Sj* myophilin-like protein (Peng et al., 2008), P7 antigen (Xu et al., 2011), 14-3-3 (Zheng et al., 2007), etc.
	Genomics	Berriman et al. (2009)	Sm200, Sm12.8, Sm43.5, Sm127.9, Sm18.9, Sm16.5 (Carvalho et al., 2011)
		The *Schistosoma japonicum* Genome Sequencing and Functional Analysis Consortium (2009)	Sj1TR, Sj4TR, Sj7TR (Angeles et al., 2011)
	Immunomics	Zhong et al. (2010)	*Sj*LAP and *Sj*FBPA (Zhong et al., 2010)
Antigen-based immunodiagnosis	Immunomics	Lu et al. (2012b)	protein BUD31 homolog, ribonuclease, SJCHGC06971 protein and SJCHGC04754 protein
PCR-based molecular method	Genomics	The *Schistosoma japonicum* Genome Sequencing and Functional Analysis Consortium (2009)	SjCHGCS19 (Guo et al., 2012)
Metabolic biomarkers discovery	Metabonomics	Balog et al. (2011)	Dimethylamine, Hippurate, PAG, etc. (Balog et al., 2011)

### ANTIBODY-BASED, INDIRECT IMMUNOLOGICAL TECHNIQUES

Indirect immunodiagnostic assays use a range of immunological methods, such as circumoval precipitin test (COPT), indirect hemagglutination assay (IHA), dipstick dye immunoassay (DDIA), and enzyme-linked immonosorbent assay (ELISA; Zhu, 2005), etc. in order to detect schistosome-specific antibodies. Immunodiagnosis based on the detection of antibodies is relatively repeatable, sensitive, tractable, and time-saving compared with traditional etiological methods. Early indirect immunodiagnosis mainly relied on the crude extracts of worm components, like microsomal extract, gut-associated polysaccharide, non-fractionated extracts of eggs, etc. (Doenhoff et al., 2004) as diagnostic antigens. However, the usage of crude antigens frequently poses a cross-reaction problem because of those components within the crude antigens shared with other, irrelevant pathogens (Jin et al., 2010). Besides, antibody-based serological assays are not quantitative and usually fail to discriminate between previous exposure and current infection. Although it is difficult to eradicate all of these innate drawbacks of these diagnostic techniques, they could be alleviated to some extent by the use of selected recombinant antigens (Doenhoff et al., 2004). Thanks to the enormous amount of gene sequence information procured by the transcriptomics and genomics researches, together with those screening methods including proteomics, immunomics as well as some bioinformatics tools, many promising diagnostic antigens have been identified and their immunogenicity have been testified, vastly enriching the diagnostic antigen pools for schistosomiasis.

Complementary DNA libraries represent a sort of precious transcriptomic resources. Under combined efforts of serological screening and immunological verification, schistosomal antigens with high immunogenicity could be identified in a cDNA library and finally applied to antibody-based immunodiagnosis of schistosomiasis. Zhou et al. (2010) used rabbit sera collected on day 21 post-infection in an antigenic screening of a previously constructed cercariae cDNA library of *S. japonicum* (Fung et al., 2002). From the identified positive clones, the authors finally selected Sjp40 as an antigen candidate and used time-resolved fluoroimmunoassay (TRFIA) to profile the levels of anti-Sjp40 IgG in the sera of rabbits with different days post-infection. The result showed that compared with the controls at each time interval, since 21 days post-infection, the titer of circulatory anti-Sjp40 IgG in the infected groups started to significantly increase. Thus, it was considered that Sjp40 was a potential antigen for early diagnosis of schistosomiasis.

Likewise, another group of researchers (Zhou et al., 2009) used human saliva, instead of rabbit sera, to screen a cDNA library of *S. japonicum* eggs, which resulted in the detection of Sj13. The following ELISA assay exhibited 92.50% sensitivity and 92.11% specificity of salivary IgG detection with recombinant Sj13. Given the easy-accessibility and non-invasiveness of the saliva samples, saliva/Sj13 was asserted as a promising alternative to serological test for schistosomiasis. In addition to these two examples, numerous antigens, sorted from the cDNA library established in 2003 (Hu et al., 2003), like *Sj*EFCAB (Lu et al., 2012a), *Sj*E16 (Wang et al., 2003), *Sj* myophilin-like protein (Peng et al., 2008), P7 antigen (Xu et al., 2011), etc. have been cloned and expressed in *E. coli* prokaryotic expression system even earlier and their immunogenicity and diagnostic potential were also investigated. Despite of their considerable sensitivity and specificity as diagnostic antigens, since most of them were published in Chinese journals, their significance thereby waned.

Besides cDNA libraries, schistosomal genomes stand for another kind of resources to search potential indirect diagnostic antigens for schistosomiasis. Using bioinformatics screening, another group succeeded in identifying several novel diagnostic antigen candidates from the previously sequenced *S. mansoni* genome (Berriman et al., 2009). Overall, four criteria were used in the* in silico* analysis strategy, including expression in all parasite life stage within the definitive host, extracellular or plasmatic membrane localization, low similarity to human and other helminthic proteins and presence of predicted B cell epitopes (Carvalho et al., 2011). As a result, six promising diagnostic antigens (Sm200, Sm12.8, Sm43.5, Sm127.9, Sm18.9, Sm16.5) were elicited from 13,197 transcripts described for *S. mansoni* (Berriman et al., 2009). Ensuing verification steps including 1D- and 2D-Western blotting using schistosomula antigen preparation, adult worm preparation and sera from infected mice indicated the good immunogenicity of those predicted antigens.

Furthermore, bioinformatics techniques also served as screening tools to seek antigen candidates for indirect immunological diagnosis in the *S. japonicum* genome (Angeles et al., 2011). A group of antigens termed tandem repeat (TR) proteins were targeted in this study because of their role in humoral responses known previously (Kim et al., 2001; Goto et al., 2010). Specifically speaking, TR genes were identified from *S. japonicum* genome by a program named Tandem Repeats Finder. A total of 12,657 gene sequences were analyzed and 134 genes were found to have TR regions. Four TR genes, i.e.,*Sj*1TR, *Sj*2TR, *Sj*4TR, and *Sj*7TR were selected for further research from the top 20 hits elicited by *in silico* screening based on their biochemical properties, conservation with other organisms, expression evidence, etc. Their recombinant proteins in expected size were successfully expressed and purified and ELISA was performed using sera from healthy people in endemic or non-endemic countries, post-treated individuals, stool-confirmed patients and patients of other parasitic diseases. *Sj*7TR had the highest sensitivity (85.71%), while both *Sj*1TR and *Sj*7TR had 100% specificity. More importantly, no cross-reaction with sera from patients infected by other pathogens was detected among those TR proteins. In contrast, the crude antigen of *S. japonicum*, SEA, had a higher sensitivity of 97.14% compared with the recombinant antigens but its specificity was poor (71.76%) and cross-reaction emerged with *Paragonimus westermani*, *Opisthorchis viverrini*, and* Entamoeba histolytica*-positive samples. In conclusion, *Sj*7TR was a promising candidate antigen for diagnosis purpose found by this research.

In another study, immunomics methods were also employed to uncover prospective diagnosis antigens for schistosomiasis (Zhong et al., 2010). In the beginning, more than 30 immunodominant spots were recognized by pooled sera from infected rabbits with Western blotting, 10 of which were precisely matched to the homologous 2D-gel. LC/MS-MS was then adopted to identify those 10 spots and they were found to correspond to four distinct proteins. Two of the four identified proteins that had not been investigated before, namely, *Sj*LAP, a complex of metallopeptidases and *Sj*FBPA, a central glycolytic enzyme were successfully cloned and the recombinant protein products were further applied in the diagnosis of *Schistosomiasis japonica* by ELISA, which yielded sensitivities of 98.1 and 87.8% for acute and chronic schistosomiasis with r*Sj*LAP and 100 and 84.7% with r*Sj*FBPA, respectively, whereas the specificities were 96.7% for both antigens. Moreover, both antigen ELISA assays showed more than 80% sensitivity in diagnosis of chronic schistosomiasis with a low intensity infection and significantly declined antibody titers after the treatment with PZQ. Thus, r*Sj*LAP and r*Sj*FBPA proved to be useful diagnostic antigens for *S. japonicum* infection.

### ANTIGEN-BASED DIRECT IMMUNOLOGICAL DIAGNOSIS

Early immunoassays have shown that schistosome-derived antigens, such as adult worm antigen (AWA), soluble egg antigen (SEA), and circulating antigen (CA; Zhao et al., 2012) could be released into the host circulatory system/excreta by schistosomes, which facilitates the researches on the direct immunological diagnosis of schistosomiasis. Circulating anodic antigen (CAA) and circulating cathodic antigen (CCA) are by far the most extensively used antigens for the antigen-based immunodiagnosis of schistosomiasis and methods for the detection of CAs encompass sandwich ELISA (De Jonge et al., 1988), IHA (Deelder et al., 1989), time-resolved immunofluorometric assay (TR-IFMA; De Jonge et al., 1989), magnetic bead immunoassay (Gundersen et al., 1992), reagent strips (Van Etten et al., 1994), and liquid-phase piezoelectric immunosensor (LP-PEIS; Wen et al., 2011). Compared with the two foregoing diagnostic methods, the detection of CA is more suitable for drug efficacy trials owing to its high specificity and the ability to discriminate between previous exposure and current infection. Moreover, CAA and CCA can be readily detected in urine (Van Dam et al., 2004), which is easier and less invasive to be collected than blood required for antibody detection. Nonetheless, low sensitivity (Van Lieshout et al., 1995, 2000), failing to distinguish different species (Agnew et al., 1995) are disadvantages associated with this method.

Research conducted by Lu et al. (2012b) becomes the first and currently the sole immunomics-based study in an attempt to profile CAs in *Schistosoma* spp. In this study, AWA was initially prepared using* S. japonicum* worms collected from infected rabbits and then employed to subcutaneously immunize the hyline hens so as to produce anti-AWA IgY. The IgY antibody contained in the egg yolk was purified by water-dilution and ammonium sulfate precipitation approach and characterized by ELISA and Western blotting. Afterwards, purified IgY was immobilized onto aldehyde-activated beaded agarose resin and served as a capture antibody to immunoprecipitate and enrich CAs within the sera of patients. At last, the obtained CAs were separated by 1D electrophoresis and analyzed by LC-MS/MS, which lead to the identification of four proteins, i.e., protein BUD31 homolog, ribonuclease, SJCHGC06971 protein, and SJCHGC04754 protein. The following analysis indicated that those four CAs belonged to neither CAA nor CCA and had not been reported from previous proteomics researches. CAs revealed by corresponding -omics researches, just like these four, ought to be cloned and expressed in the future and the produced mAbs could be applied for antigen detection through sandwich ELISA which stands for a promising way to overcome the shortcomings of the current direct immunodiagnostic methods.

In addition to this finished work, one recent review about proteomics at the schistosome–mammalian host interface (Wilson, 2012) also proposed several candidate antigens that can be potentially used for CA detection, including serpin, α2 macroglobulin, Am200, LMWP, as well as some MEG-2 and MEG-3 proteins secreted by eggs.

### NUCLEIC ACID AMPLIFICATION-BASED MOLECULAR METHODS

Given the existence of schistosomal DNA in the serum and other tissue samples of the host derived from dead worms, tegument shedding or inactive eggs (Xu et al., 2013), PCR-based molecular diagnosis has become a promising alternative to overcome the innate shortcomings of etiological and immunological diagnosis for schistosomiasis. Since the publication of the proof-of-concept study in 2002 (Pontes et al., 2002), PCR-based molecular diagnosis has been applied to the detection of schistosomes in multiple diagnostic field works (Pontes et al., 2003; Obeng et al., 2008; Enk et al., 2012) and several novel technologies, such as real time PCR (RT-PCR; Lier et al., 2006), PCR-ELISA (Gomes et al., 2010), and loop-mediated isothermal amplification (LAMP; Xu et al., 2010), etc. have been added to the tool assemblies in addition to conventional PCR in order to boost the detection sensitivity. Empirical evidence showed that molecular diagnosis of schistosomiasis has both high sensitivity and high specificity, enables detection during larval stage or at least before egg spawning and can use manifold materials, e.g., feces, serum (Pontes et al., 2002), plasma (Wichmann et al., 2009), and urine (Sandoval et al., 2006) as templates, all of which make it superior to the conventional diagnostic approaches. Factors that impact the result of molecular diagnosis are various and the choice of amplified products is definitely among them. However, confined by the inaccessibility to the schistosomal genome sequence, in the pre-genomic era, researchers could only select targets for PCR reactions within limited candidates. Representative examples include two different 121-bp repetitive elements in *S. mansoni* (Hamburger et al., 1991) or *S. haematobium* (Hamburger et al., 2001), *S. japonicum* 18s rDNA (Zhou et al., 2011a), highly repetitive retrotransposon *Sj*R2 (Laha et al., 2002), mitochondrial NADH1 gene (*nad1*) of *S. mansoni* (Jannotti-Passos et al., 1997) and *S. japonicum* (Lier et al., 2008), etc.

As early as 2009, 25 novel retrotransposons had been identified along with the decoding of the genome of* S. japonicum* (The *Schistosoma japonicum* Genome Sequencing and Functional Analysis Consortium, 2009). Two years later, a large scale selection had been conducted in order to seek suitable target sequences among those 25 retrotransposons to achieve the sensitive and specific diagnosis of *S. japonica* (Guo et al., 2012). To begin with, primer pairs were designed for the 25 candidates with *Sj*R2, a previously described molecular diagnostic target (Xia et al., 2009; Xu et al., 2010), serving as a positive control. A series of diluted genomic DNA of *S. japonicum* were used as the templates in a PCR assay to amplify the target fragments and judge the sensitivity of those 25 candidates. The result showed that in addition to *Sj*R2, a new non-LTR retrotransposon named *Sj*CHGCS19, with the PCR product of 303-bp in length, had high sensitivity for detecting *S. japonicum* DNA. Coincidentally, bioinformatics research showed that both *Sj*CHGCS19 and *Sj*R2 have higher genome proportions, repetitive complete copies and partial copies and more active ESTs compared with other candidates in the *S. japonicum* genome, which may also outline the general features of ideal targets for PCR diagnosis of schistosomiasis. Besides, further researches showed that *Sj*CHGCS19 has good specificity except for a cross-reaction with *S. mansoni* and it is also an ideal PCR product for the early diagnosis of schistosomiasis as well as the evaluation of chemotherapy. At last, the clinical utility of *Sj*CHGCS19 was also tested by diagnosing 43 human serum samples from patients of *S. japonica* and 51 serum samples from healthy individuals. As a result, the sensitivity of 97.67% and the specificity of 96.07% fully validated the effectiveness of the *Sj*CHGCS19-based molecular diagnosis of schistosomiasis.

Parasite-derived miRNAs, comparable to genomic DNA, are also present in multiple biofluids of the host, such as plasma, serum, urine, and saliva (Cortez et al., 2011), which implies their potential as biomarkers for the molecular diagnosis of schistosomiasis. Besides, in response to the invasion of pathogens, some characteristic changes of the host’s miRNAs profile might appear (Delić et al., 2011), which can also serve as hints of infection event. Nowadays, the available sequences of schistosomal miRNAs, in association with those approaches established for the profiling of miRNAs, e.g., stem-loop RT-PCR, miRNA array, and high-throughput sequencing (Vaz et al., 2010) have paved the way for the future miRNA-based molecular diagnosis of schistosomiasis. For detailed information about the diagnostic and therapeutic value of parasite miRNA, see review (Manzano-Román and Siles-Lucas, 2012).

### METABONOMICS-BASED BIOMARKER DISCOVERY

As mentioned above, early schistosomal metabonomics focused on the dynamics of biochemical responses associated with *S. japonicum* or *S. mansoni* infection in multiple biofluids of rodent models. Corresponding progresses made thus far have been reviewed in several papers (Legido-Quigley, 2010; Wang et al., 2010a; Utzinger et al., 2011). To sum up, the metabolic fingerprint of rodents in response to schistosomes infection can be concluded as: reduced levels of the tricarboxylic acid cycle intermediates, stimulated glycolysis, disturbed amino acid metabolism, disturbances in the gut microbiota as well as a phenomenon unique to *S. japonicum* infection, i.e., the inhibition of short-chain fatty acids. Despite the considerable progresses achieved in rodent models, clinical diagnosis of schistosomiasis based on metabonomics still lags behind other -omics methods, because after all, the physiological mechanisms between rodents in the laboratories and humans are far from similar. However, the newly published metabonomic work about human schistosomiasis (Balog et al., 2011) has laid a solid foundation for the clinical application of metabonomic outcomes in the future. Prospective molecular signatures of human *S. mansoni* infection found by this study were primarily related to changes in gut microflora, energy metabolism, and liver function, in line with the earlier findings in experimental animals.

## FUTURE DIAGNOSTIC TOOLS OF SCHISTOSOMIASIS AND ADVICE TO BETTER HARNESS -OMICS ACHIEVEMENTS

In our view, future diagnostic tools of schistosomiasis ought to follow three main developmental trends: (1) Field-applicability, test kit protocols should be robust, easy to follow, and produce results within a few minutes without the aid of extra instruments. (2) Non-invasiveness, diagnostic tools should detect specific biomarkers from easy accessible biofluids, e.g., sweat, urea, or saliva. (3) High-throughput, diagnostic kits in the next generation should integrate multiple probes and enable parallel identification of different pathogens.

“-Omics” studies result in a treasure trove of molecular data on genes, proteins, and metabolites. However, identification of effective diagnostic biomarkers from these data pools requires bioinformatics tools based on certain criteria. Further summarization of the general features shared by those currently available diagnostic targets will undoubtedly help us optimize the algorithms for biomarker selection and thereby better utilize the information generated by -omics studies. Besides, some molecular candidates of great diagnostic value are able to be sorted directly by -omics tools based on either their positive interactions with certain biofluids of patients or the changes of their presences or concentrations in response to parasite infection. Schistosomal samples and/or biofluids of patients used in such researches should be chosen and combined according to the prospective utility of a diagnostic tool. Immunomics and metabonomics studies using distinct biological samples should be rigorously designed and carried out in future, in an attempt to seek more diagnostic biomarkers that satisfy variant requirements, e.g., to distinguish infection by different *Schistosoma* species and/or with different worm burden, to discriminate between current infection and previous exposure, etc. Among all available body fluids to date, priority should be given to those that can be sampled without invasive procedures in order to achieve a rapid and safe diagnosis. Last but not least, we should also pay attention to the achievements of those newly established -omics tools, so as to enrich our arsenal of schistosomiasis diagnosis with innovative biomarkers.

Progress in the control and prevention of parasitic diseases is expected to be more rapid and efficient thanks to the achievements of “-omics” researches, benefitting the health of humans and animals alike.

## Conflict of Interest Statement

The authors declare that the research was conducted in the absence of any commercial or financial relationships that could be construed as a potential conflict of interest.
